# Juridification of maternal deaths in Ethiopia: a study of the Maternal and Perinatal Death Surveillance and Response (MPDSR) system

**DOI:** 10.1093/heapol/czaa043

**Published:** 2020-06-28

**Authors:** Andrea Melberg, Lidiya Teklemariam, Karen Marie Moland, Henriette Sinding Aasen, Mitike Molla Sisay

**Affiliations:** c1 Centre for International Health, University of Bergen, Årstadveien 21, N-5007 Bergen, Norway; c2 O’Neill Institute for National and Global Health Law, Georgetown University Law Center, USA; c3 Faculty of Law, University of Bergen, Norway; c4 School of Public Health, College of Health Sciences, Addis Ababa University, Ethiopia

**Keywords:** Ethiopia, maternal health, juridification, indicators, malpractice, MPDSR, multi-sited ethnography

## Abstract

Juridification of maternal health care is on the rise globally, but little is known about its manifestations in resource constrained settings in sub-Saharan Africa. The Maternal and Perinatal Death Surveillance and Response (MPDSR) system is implemented in Ethiopia to record and review all maternal and perinatal deaths, but underreporting of deaths remains a major implementation challenge. Fear of blame and malpractice litigation among health workers are important factors in underreporting, suggestive of an increased juridification of birth care. By taking MPDSR implementation as an entry point, this article aims to explore the manifestations of juridification of birth care in Ethiopia. Based on multi-sited fieldwork involving interviews, document analysis and observations at different levels of the Ethiopian health system, we explore responses to maternal deaths at various levels of the health system. We found an increasing public notion of maternal deaths being caused by malpractice, and a tendency to perceive the juridical system as the only channel to claim accountability for maternal deaths. Conflicts over legal responsibility for deaths influenced birth care provision. Both health workers and health bureaucrats strived to balance conflicting concerns related to the MPDSR system: reporting all deaths vs revealing failures in service provision. This dilemma encouraged the development of strategies to avoid personalized accountability for deaths. In this context, increased juridification impacted both care and reporting practices. Our study demonstrates the need to create a system that secures legal protection of health professionals reporting maternal deaths as prescribed and provides the public with mechanisms to claim accountability and high-quality birth care services.

**Key Messages**

- Litigation is increasingly seen by community members as a way to obtain accountability for maternal deaths
- Fear of litigation influences birth care provision and may encourage defensive medicine
- Health workers develop strategies to avoid personalized accountability for deaths.

## Introduction

In February 2019, the Ethiopian Society of Obstetrics and Gynaecologists (ESOG) held their annual meeting in one of Addis Ababa’s conference venues. In the market space outside the plenary hall, conference sponsors had set up their small booths. Among pharmaceuticals and providers of medical supplies and diagnostic services, an insurance company joined the conference for the first time, advertising a professional indemnity insurance. During the conference, several of the conference attendees expressed how the first-time attendance of an insurance firm was symptomatic of an increased presence and importance of medico-legal issues in clinical life.

Juridification of healthcare provision seems to be an increasing global trend (Aasen *et al.*, 2014; Van Belle *et al.*, 2018). In addition, there is growing recognition of law being an important health determinant (Gostin *et al.*, 2019). However, little is known about the manifestations of juridification in resource constrained health systems and how the dynamics between juridification and the implementation of health policies play out in concrete healthcare settings in sub-Saharan Africa. The current article will contribute to fill this gap by studying these dynamics in the context of maternal care in Ethiopian. Studies have identified fear of blame and litigation among healthcare professionals as one of the reasons for not reporting maternal deaths (Melberg *et al.*, 2019). Using the Ethiopian Maternal and Perinatal Death Surveillance and Response (MPDSR) system as an entry point, this article therefore seeks to investigate the dynamics between juridification processes in maternal health and the provision of birth care in Ethiopia. We have a particular emphasis on maternal deaths, and the responses these deaths engender. Drawing on our empirical findings, we reflect on the consequences of juridification—particularly in terms of MPDSR implementation and maternal healthcare provision—in the Ethiopian healthcare system.

### Juridification

Juridification has been described by Habermas (1987) as ‘[…] the tendency toward an increase in formal (or positive, written) law that can be observed in modern society’ (p. 359). Blichner and Molander’s (2008) characterize juridification as a process with five dimensions: shifts into constitutive regulations, the proliferation of legal regulations, conflict solving with reference to law, increased judicial power and lastly increased legal framing. Others have analysed specific processes, consequences and implications of juridification and judicialization (litigation and court proceedings) in different social fields, including health care (Gloppen and Yamin, 2011; Aasen *et al.*, 2014). In global health, juridification is often associated with the ‘health litigation pandemic’ in the Americas and the increasing number of malpractice litigations in South Africa (Vargas-Peláez *et al.*, 2014; Pepper and Slabbert, 2011). In maternal health, the role of formal law is probably most visible in the contested access to safe and legal abortions throughout sub-Saharan Africa (Blystad *et al.*, 2019).

Studies addressing juridification of health care in low-income countries in sub-Saharan Africa are few in numbers and focus mostly on the regulation of private for-profit providers (Doherty, 2015). More than a decade ago, Harrington (2004) stated that in Tanzania there seemed to be ‘no widespread perception of litigation as a means of providing for the accountability of the agents and institutions of the state including the great majority of medical professionals who worked for it’. Reasons include general poor access to legal services, a limited number of legal professionals, predominantly public health care with relatively few private for-profit providers and a population that is not used to be rights claimants. However, throughout sub-Saharan Africa, there has been a burgeoning of private for-profit healthcare providers the past years, especially in urban centres (Doherty, 2015). International and national public discourses on rule of law and accountability for health the last decades indicate an increased utilization of legal strategies also in low-resource settings of sub-Saharan Africa (Harrington, 1998).

### Responses to maternal deaths in Ethiopia

In Ethiopia, like other low resource settings, maternal deaths still constitute a considerable risk for women giving birth inside and outside of health facilities. It is estimated that ∼13 000 women die from pregnancy-related causes every year (Ethiopian Public Health Institute, 2017). However, maternal deaths figures in Ethiopia, like in many other settings, are highly uncertain. This is associated with the technical difficulties in producing maternal mortality data and their politicized nature (Wendland, 2018). Maternal deaths are classified as a public health emergency in Ethiopia, and the government proclaims that ‘No mother should die while giving birth’. Key maternal health indicators such as maternal mortality ratio, skilled birth attendance and total fertility rates have been dramatically improved the past decades (Assefa *et al.*, 2017). These achievements may partly be attributed to the strong Ethiopian state, characterized as ‘developmental authoritarian’ in which the state exerts control over socio-economic development in all facets of society (Matfess, 2015). This has implications both for the state’s capacity to roll out policies, and for the knowledge produced about maternal health challenges (Østebø *et al.*, 2018). Health workers and bureaucrats have been reported to experience a strong pressure not to report maternal deaths as this would taint government efforts to prevent these deaths (Melberg *et al*., 2019).

To accelerate maternal mortality reduction in Ethiopia, a country wide MPDSR system was set up from 2013 to identify all maternal and perinatal deaths occurring in the country, inside and outside healthcare facilities (Abebe *et al.*, 2017). Reviews were to be conducted at all levels of the health system, from the health centre and hospital levels to the regional and federal levels with the aim to identify underlying causes of deaths, and to propose and implement remedial actions to prevent similar deaths from reoccurring.

International and national MPDSR guidelines explicitly state that death reports and reviews conducted within MPDSR should not be used as grounds for health worker punishment. Still, after 5 years of implementation, only about 10% of the expected maternal deaths occurring in the country were reported in the MPDSR system in 2017 (Ethiopian Public Health Institute, 2017). At the same time, the number of malpractice accusations directed towards Ethiopian obstetricians are on the rise (Wamisho *et al.*, 2015; Teklu *et al.*, 2017), and obstetricians accused of malpractice are frequently being questioned by the police or an attorney. Several report to have been imprisoned for shorter or longer periods of time because of such allegations (Teklu *et al.*, 2017).

At the time of that the study was conducted, the Ethiopian Food, Medicines and Healthcare Administration and Control Authority (Council of Ministers Regulation No. 299/2013) monitored healthcare practice and practitioners. The Federal Health Professionals Ethics Committee (FHPEC) had the power to investigate and decide on complaints of substandard health. It was also their responsibility to propose administrative measures, including permanent and temporary revocation of license, suspension, warning, order for additional training and prohibitions from providing certain services (Wamisho *et al.*, 2015). While common courts decided on the civil and criminal liabilities of providers, they frequently relied on the reasoning and advice from the FHPEC. According to an analysis of the 125 decisions FHPEC passed between 2011 and 2018 (unpublished data, article under review), obstetricians were the group of clinicians most frequently accused of malpractice. In more than half of all complaints, the outcome of the incidents was death, mostly maternal deaths.

## Methods

This study is based on an ethnographic, multi-sited fieldwork (Marcus, 1995) carried out by the first author, a Norwegian medical doctor in Addis Ababa and in the surroundings of a medium-sized town with ∼40 000 inhabitants in July/August 2018, October/November 2018 and February 2019. Regional institutional review boards (anonymous) ethically approved the study.

The initial aim of the study was to explain the current levels of low reporting of maternal deaths in the MPDSR system. Our research problem and our pre-conceptions from previous health system research carried out in Ethiopia influenced the strategies employed in data generation and analysis. We wanted to identify the local MPDSR practices which produce data on maternal deaths. Since the data flow between the local and national health system levels, we deemed it appropriate to use interviews and document analysis of maternal death reporting and reviews at community, health facility, woreda (district) and zonal (sub-regional) level as our primary approach.

The first author also recorded on a daily basis her observations and notes from informal conversations conducted in the communities and in the health facilities, woreda and zonal offices where the study participants worked. Juridification of maternal deaths was not an initial study objective but very early in the fieldwork it emerged as a major concern of the study participants. Aspects of juridification were therefore gradually given more attention during the observations, in informal conversations with health workers and in formal interviews. In the last period of fieldwork, the first author gained invaluable contextual knowledge while attending the annual meeting of the Ethiopian Society of Obstetrics and Gynaecologists.

Health system responses and perspectives on juridification remained at the core of the study, and we did not seek to include participants from the legal system. The study only included public health facilities, given the predominantly public nature of the Ethiopian health system. One might expect manifestations of juridification to be more pronounced in private facilities utilized by a generally wealthier and more educated patient population.

The first author conducted a total of 46 in-depth interviews: 11 with primary caregivers who had experienced perinatal deaths, 5 with men who had lost their partner to a maternal death, 4 with health extension workers, 7 with health workers working in general- and referral hospitals (health officers, medical doctors, nurses, midwives), 13 with health workers working in health centres (nurse, midwives) and 6 with health bureaucrats responsible for MPDSR implementation at woreda (county), zonal and federal levels. The interviews centred around what kind of responses maternal and perinatal deaths would trigger, with a particular emphasis on how maternal deaths were translated into registries and reports. The interviews were conducted in Amharic or English. In the Amharic interviews, an Ethiopian research assistant trained in public health translated. The interviews lasted from 30 to 105 min, were tape-recorded and transcribed verbatim. Amharic transcripts were translated to English.

Maternal deaths constitute a sensitive topic in Ethiopia, and the study participants tended to expect the interviewer to make a critical assessment of their work. This was a main obstacle to obtain access to the field and information about care and reporting practises in cases of maternal deaths. Managers are regularly assessed based on the numerical performance of their health facility, and maternal deaths are an important indicator (Melberg *et al*., 2019). However, the first author’s identity as an outsider in Ethiopia and the Ethiopian health system, and her professional identity as a health worker proved valuable in gaining trust, particularly among clinicians and medical doctors working in the health bureaucracy. Formal informed consent was obtained from all participants. As we recognize that medico-legal issues surrounding maternal and perinatal deaths constitute sensitive issues for bereaved families, front-line health workers and health bureaucrats, we have chosen to refrain from giving more details on the study participants and the study locations to protect the anonymity of the study participants.

After initial analysis during fieldwork, the data were analysed using thematic content analysis. Analysis was conducted on fieldnotes and translated English transcripts by the first author, with reference to the Amharic transcripts for clarification when necessary.

## Findings

In the following section, we explore how next of kin allocate blame for maternal deaths and see the legal system as a way to claim accountability for maternal deaths. We also document how medico-legal issues impact the everyday life of health workers, and the choices they make when reporting maternal deaths and providing birth care.

### ‘I Think it was their failure’

Throughout observations and interviews there seemed to be a common understanding among next of kin that maternal deaths were to be prevented by facility births. Health workers, by contrast, classified many maternal deaths as non-preventable. Both next of kin and health workers expressed how maternal deaths would fuel long-term conflicts and distrust between health institutions, communities and political leadership. Faced with maternal deaths, it seemed important for both groups to identify failures in treatment and the individuals responsible for these, like this widowed husband explained:

*I asked them why they are watching while the (blood) pressure is going up and they should take the baby out. At that time, I asked them to let me take her to another place.*

*Yes, when they refused, I was confused and wanted to take her to Addis Ababa Black Lion Hospital (National referral hospital). I had arguments with the health workers … There was one person who works at the hospital I was very much fond of him, but now I don’t even talk to him on the road; you understand. I asked him to let me take her to another place.*

In cases where next of kin expressed discontent with the services provided during childbirth ending with a maternal or perinatal death, blame was directed towards individual health workers, not health institutions or the lack of adequate resources. Health worker negligence was perceived as the central cause of deaths. Either the health worker did not care properly for the woman or baby, or they were not knowledgeable enough to recognize or manage the complication. Delays before receiving care and delayed referral to a higher-level facility was seen as major causes of death. All of these reasons were present in the tale of Yonas, a civil servant whose wife died in the aftermath of an unsafe abortion. According to him, the nurses present in the gynaecological ward were too busy gossiping and did not care to examine his wife properly upon arrival this early Saturday morning. Even if he repeatedly summoned them, it was only when his wife lost consciousness in the afternoon that the nurses intervened:

*The time she stayed there (at the hospital) and the service she got is not comparable. We reached there at 9:00 am. If she had gotten the service and if they evacuated it (removal of foetal products from the uterus) starting at that time, she could have been cured. She was bleeding the whole day and they evacuated it (the uterus) later, but if they did it earlier, they could save her. Not doing what was required, they referred her (to another hospital) at the end.*

Like Yonas, many next of kin that experienced what they saw as insufficient treatment had initial thoughts of going to the police or the courts and were encouraged by neighbours and family members to do so. This was also the case in the small town situated outside the capital city of Addis Ababa. When bringing up the matter during informal conversations in the community, perceived benefits of presenting the case to the legal system included procuring information on what exactly happened and to force the health workers in question to be more careful and to take lessons. However, none of the participants had actually taken the case further in the legal system. Reasons included lack of concrete evidence, a decision to focus on the future and the feeling that going further would be useless as health workers and hospitals were covering up deaths. The narrative of another man who lost his wife illustrates this point:

*She was in good health. Her uterus ruptured during labour. Had it been known earlier and (they had) made her deliver by surgery then she would not have bled this much and the ruptured part would have been seen and repaired so she could have been saved. I think it was their failure…. Yes, it was a medical error. The lady who evaluated her should have not proceeded with the labour. She should have made her deliver with surgery. Everyone knows this but as they are workers of one hospital, they keep cases confidential and confidentiality should be kept. Yes, that is why they covered it up. I dropped the case as I was helpless. It is to cover up the lady’s mistake. Even the specialist doctor was very disappointed as this happened in a time at which it is said that no mother should die. He did not show up at work for 15 days.*

Besides engaging with the legal system, even the participants that strongly expressed their discontent with the health care received, felt unsure about where to address complaints about substandard treatment and care. Some had engaged in quarrels directly with health workers present at the time of death, whereas others found complaining to be pointless as health workers would soon go back to old habits. Most family members were never given an explanation of the reason of death. None had been interviewed about the events leading up to the death, as prescribed by the MPDSR system.

### ‘You might protect yourself’

Health workers and bureaucrats interviewed highlighted how the MPDSR system was not set up to trace individual health worker blame or to be used as a basis for litigation, but rather should focus on collective responsibility and future improvement. This strongly contrasted with the descriptions of what would happen to health workers in the aftermath of maternal deaths. Community members and health workers referred to cases they knew of where health workers had been accused of causing maternal deaths, and many mentioned how such cases were rapidly increasing in numbers due to increased media coverage and community awareness. Whereas medical doctors seemed particularly concerned with the more formal legal accusations, lower-level health workers worried primarily about informal sanctions imposed by the local political leadership or health administration. Birth care providers expressed a strong concern that patients and the media found all complications to be the responsibility of the providers, not taking into consideration unpreventable deaths, and that women would present too late at the health facility for the health workers to intervene. Similarly, health workers expressed how the legal system would not take into account the infrastructural challenges and resource scarcity leading to maternal deaths, such as lack of ambulances, oxygen and blood:

*Even, there are times when we don’t have blood and the woman dies, especially in remote areas, not near big cities. Relatively this is a big city. But if you go further and further, blood might not be available easily. So, you watch, while you are watching the woman could die. So, in such cases the legal bodies do not say that there is no blood because the government doesn’t, or is unable to provide blood, they don’t say like that. Just, what have you done, that is the question to you, you have to tell them the problem and at some point, you might protect yourself.*

Health worker litigation after maternal death was a delicate issue in the professional community, but some particular cases seemed to be well known among obstetricians. Although we identified quite a number of these, the individuals involved were reluctant to talk about these processes. Several study participants had been questioned by the police after a maternal death and experienced the process as distressing and time-consuming. Many reported how they had colleagues that had been picked up by the police at their workplace and jailed for days after a maternal death. A few of these cases were brought to court as first-degree murder, not medical negligence or malpractice. Obstetricians expressed a frustration that court cases pointed to individual senior specialist, not to the hospital as an institution. These individual consequences were discussed as lack of health worker protection in the Ethiopian health system. Under these circumstances, doctors sensed a growing scepticism among younger colleagues to go into clinical obstetrics:

*Yes, because if it is not your fault even, when a mother dies you suffer. Just, here is meeting, here is meeting, somebody calls you, somebody phones the woreda for you, or somebody from regional office come and talk and you get disturbed. Just that stress. Usually many people don’t want to be near gynaecology and obstetrics because of that… It is very political. There are many gynaecologists that are in jail. They do what must be done, but people are very, very aware. It is not very aware, more than aware even. And they just find some problems; it could just be some delaying when the gynaecologist is called to travel from his home to the ward, then if something happens, they just accuse him. And with that, they just call him to the court.*

During the closing session of the annual meeting of the gynaecologists’ professional association, a leading gynaecologist took the stage to plead the audience to provide economic and moral support to a fellow colleague. The colleague was imprisoned after a maternal death taking place under his responsibility and was currently facing health problems needing costly treatment. Gynaecologists at the conference were upset about his situation and seemed preoccupied with possible ways in which they themselves could obtain personal and financial protection if maternal deaths occurred. Many referred to the newly introduced indemnity insurance mentioned in the introduction of this article. While some conference participants saw the necessity of such insurance, especially when working in the private sector, others said that the insurance would not be of help given the lacking malpractice legislation in the country. The salesperson present at the conference admitted that they had not sold many insurances, but that she considered it a growing marked.

The fear of being accused of maternal deaths also resulted in what clinical providers labelled as defensive referral and medical practices. In informal discussions with several senior gynaecologists, there seemed to be a consensus that dying mothers were ‘dumped’ on tertiary hospitals by other hospitals to avoid accountability measures. One gynaecologist working in a referral hospital exemplified this dumping by explaining how women with extra uterine pregnancy were referred directly to them without any required surgical procedure to stop the bleeding. To his understanding, health workers chose not to intervene due to the fear of medico-legal consequences, and by doing so, patients did not receive potential life-saving treatment. Hence, the fear of accountability may result in unnecessary deaths.

## Discussion

Our study documents problematic aspects relating to implementation of the MPDSR system in Ethiopia. In particular, the manner in which criminal law procedures are used against health workers cause problems both from the perspective of accused individuals and from a health system perspective. In the following, we first discuss the dynamics between juridification processes and MPDSR implementation. Second, we reflect on the wider consequences of juridification in resource constrained health systems.

### Juridification and MPDSR implementation

The MPDSR system is designed to improve data on maternal deaths, to increase accountability for maternal health and to advance quality of pregnancy and birth care (Bandali *et al.*, 2016). The creation of a no-blame culture has been put forward as a key strategy to achieve these outcomes (Smith *et al.*, 2017). According to implementation guidelines, data collected as a part of the MPDSR system are not to be used in litigation (WHO, 2016). However, as this study documents, accusations of individual health workers, criminal procedures, imprisonment and litigation have become real threats in cases of maternal deaths.

The MPDSR system aims to improve accountability for maternal health. This article has highlighted how the strong public discourse on zero maternal deaths in a health system that is still in many ways incapable of preventing these deaths, fuels distrust between communities and health workers. There were insufficient mechanisms and channels where patients could declare their discontent with health services. The judiciary system thus became one of few possibilities to claim accountability for services. However, maternal deaths were not interpreted as an expression of health system failure or as a failure of the government to provide maternal health care. Rather, it was portrayed as a result of individual health worker misconduct. Accordingly, health workers, especially medical doctors, increasingly perceived themselves as individual legal subjects and viewed their personal decision-making in the light of possible criminal procedures.

By identifying and reviewing all maternal deaths, health workers and bureaucrats produce knowledge about policy implementation through the categorization of complex realities into countable events and indicators (Merry, 2011). Data on maternal deaths are therefore not neutral (Adams, 2016; Wendland, 2018). Maternal mortality metrics are also closely tied to accountability and governance, as they influence the allocation of resources, the nature of political decisions and the assessment of which countries prioritize maternal health (Merry, 2011). The reliance numbers to achieve accountability has catalyzed what Strathern (2000) names an ‘audit culture’, also within the field of maternal health. The increased push for numbers and accountability in maternal health are interlinked with juridification processes. In an increasingly juridified context, health workers experienced a growing tension between the obligation to report all deaths in the MPDSR system, and the fear to reveal failures in services provision. As previously reported (Melberg *et al*., 2019), they engaged in efforts to deflect responsibility for maternal deaths by omitting death from their reports, by redefining maternal deaths as non-maternal deaths, and by reporting deaths as unpreventable.

### The wider consequences of juridification

In a country with extremely limited resources available for both health and justice, we question whether the increased juridification documented in our study is productive in improving health and well-being, or whether it rather draws attention and resources away from the provision of quality birth care. In South Africa, the cost of rising medical malpractice claims is said to affect the state’s ability to fund the public healthcare system, and to negatively affect health equity (Pepper and Slabbert, 2011; Malherbe, 2012). Juridification and judicialization might also affect equity. As reported from Tanzania, litigation can be understood as an accountability mechanism instigating improved service delivery but might result in a further skewing of resources towards the wealthy having access to the judiciary system (Harrington, 2004).

Health workers in our study engaged in what they themselves labelled defensive practice. Defensive medicine is often seen to arise from perceived or actual threat of legal action (Bassett *et al.*, 2000). Although litigations remain rare in the Ethiopian context, they cause health worker distress and affect their clinical practices. It has been written extensively on defensive medicine in high-income countries, where it is typically portrayed as health workers ordering medically unnecessary tests and procedures to protect themselves against potential lawsuits (Tancredi and Barondess, 1978). In maternal health, the increasing caesarean section rates globally have been linked to health workers fearing blame in cases of poor maternal and foetal outcomes (Fuglenes *et al.*, 2009; Betrán *et al.*, 2018). To the contrary, obstetricians in Ethiopian portray defensive medicine as *not* intervening with required procedure with the risk of a maternal death in mind, and rather refer women to the next level of care to avoid blame, as previously also reported from Burkina Faso (Melberg *et al.*, 2016).

Obstetricians in Ethiopia express how they as a professional group are not granted fair treatment in situations of malpractice accusations. Malpractice insurance is portrayed as a means to gain personal protection from financial consequences of such accusations (Teklu *et al.*, 2017). Regulations surrounding medical malpractice in Ethiopia are scarce. Putting in place a more balanced legal regulation including fair treatment of health providers is urgent. Further legal development and implementation of appropriate accountability mechanisms are needed in order to re-establish a culture where doctors are able to do their work according to acceptable professional standards and norms of conduct, knowing that such norms will protect them against unreasonable liability claims. Such regulations will secure both good medical practice favouring patients and communities and fair treatment of health providers. However, legal development is not enough. As Bassett *et al.* (2000) note, defensive medicine remains ‘a complex social product [that] will require an equally complex social solution’ (p. 534), requiring development of professional standards and prioritization mechanisms.

## Conclusion

Community and health system responses to maternal and perinatal deaths are increasingly juridified in Ethiopia. The manifestations of juridification include accusations of criminal liability in cases of maternal deaths, and an increased legal framing of and influence on birth care provision. Such legal processes, perceived by the professional community as highly unfair, seem to impede effective MPDSR implementation and be counter-productive in terms of health outcomes. Legal procedures and cases against individual doctors draw attention and resources away from the provision of quality birth care in the Ethiopian setting. Based on the study findings, there seems to be a need both for measures to safeguard health professionals providing birth care, but also to provide communities with possibilities and mechanisms through which public accountability for services and high-quality birth care can be claimed.
